# Comparison of neuropsychiatric symptoms and diffusion tensor imaging correlates among patients with subcortical ischemic vascular disease and Alzheimer’s disease

**DOI:** 10.1186/s12883-017-0911-5

**Published:** 2017-07-28

**Authors:** Min-Chien Tu, Wen-Hui Huang, Yen-Hsuan Hsu, Chung-Ping Lo, Jie Fu Deng, Ching-Feng Huang

**Affiliations:** 1 Department of Neurology, Taichung Tzu Chi Hospital, Buddhist Tzu Chi Medical Foundation, No. 88, Sec. 1, Fengxing Rd., Tanzi Dist., 427 Taichung City, Taiwan; 2 School of Medicine, Tzu Chi University, Hualien, Taiwan; 3 Department of Psychology, National Chung Cheng University, Chiayi, Taiwan; 4 Department of Radiology, Taichung Tzu Chi Hospital, Buddhist Tzu Chi Medical Foundation, Taichung, Taiwan

**Keywords:** Behavioral and psychological symptoms of dementia, Subcortical ischemic vascular disease, Alzheimer’s disease, Neuropsychiatric inventory, Diffusion tensor imaging, Corpus callosum, Apathy, Psychosis

## Abstract

**Background:**

The causes of behavioral and psychological symptoms of dementia (BPSD) vary according to the dementia subtype and associated neuropathology. The present study aimed to (i) compare BPSD between patients with subcortical ischemic vascular disease (SIVD) and Alzheimer’s disease (AD) across stages, and (ii) explore the associations with diffusion tensor imaging (DTI) in the corpus callosum (CC) and other major fibers.

**Methods:**

Twenty-four patients with SIVD and 32 with AD were recruited. Four domains of the Neuropsychiatric Inventory (NPI) (hyperactivity, psychosis, affective, and apathy) and two DTI parameters [fractional anisotropy (FA) and mean diffusivity (MD)] within the genu, body (BCC), and splenium (SCC) of the CC and other major fibers were assessed.

**Results:**

Overall, the patients with clinical dementia rating (CDR) 1 ~ 2 had higher scores in apathy domain than those with CDR0.5. Among those with CDR1 ~ 2, SIVD had higher scores in apathy domain than AD. MD values in the BCC/SCC were positively correlated with total NPI score and psychosis, hyperactivity, and apathy domains. FA values in the SCC were inversely correlated with total NPI score and psychosis domain. The correlations were modified by age, the CASI, and CDR scores. Stepwise linear regression models suggested that FA value within the left superior longitudinal fasciculus predicted the hyperactivity domain. MD value within the SCC/left uncinate fasciculus and FA value within the GCC/left forceps major predicted the psychosis domain. MD value within the right superior longitudinal fasciculus and CDR predicted the apathy domain. Further analysis suggested distinct patterns of regression models between SIVD and AD patients.

**Conclusion:**

White matter integrity within the BCC/SCC had associations with multi-domains of BPSD. Our study also identified important roles of regions other than the CC to individual domain of BPSD, including the left superior longitudinal fasciculus to the hyperactivity domain, the left uncinate fasciculus/forceps major to the psychosis domain, and the right superior longitudinal fasciculus to the apathy domain. The neuronal substrates in predicting BPSD were different between SIVD and AD patients. Of note, apathy, which was more profound in SIVD, was associated with corresponding fiber disconnection in line with dementia severity and global cognition decline.

**Electronic supplementary material:**

The online version of this article (doi:10.1186/s12883-017-0911-5) contains supplementary material, which is available to authorized users.

## Background

Behavioral and psychological symptoms of dementia (BPSD) are defined as “symptoms of disturbed perception, thought content, mood, and behavior frequently occurring in patients with dementia” [1]. Recent epidemiological studies have suggested that BPSD may be responsible for premature institutionalization, caregiver stress and use of health care resources [2, 3]. While the reported prevalence of individual symptoms varies, apathy, depression, anxiety, irritability, aggression/agitation and delusions are commonly reported BPSD [4, 5]. Patients with dementia often exhibit a combination of these symptoms and tend to present with changed behaviors at a certain stage of disease. This suggests that underlying organic brain structural changes and distinct neurobiological properties may be responsible for the pathogenesis of BPSD. The presentations of BPSD and their relationships with the severity of dementia have also been reported to vary according to the subtype of dementia, although the results have been inconsistent [6, 7]. Studies comparing BPSD between patients with vascular dementia (VaD) and Alzheimer’s disease (AD) have reported varying results with regards to frequency [8] and according to care setting [9], or even no apparent differences [6]. This inconsistency in results may partially be explained by the fact that VaD is comprised of a group of heterogeneous pathologies. Moreover, patients with dementia with advancing age are more likely to have both AD and VaD pathology, therefore confounding their relevant clinical presentations.

To investigate the neurobiological basis of BPSD, the diagnosis of subcortical ischemic vascular disease (SIVD), with defined pathological properties and involved regions, provides an opportunity to further probe the impact of subcortical lacunes and white matter changes with regards the clinical presentations in a more straightforward manner [10]. Some studies comparing patients with SIVD and AD have reported a trend of a higher prevalence of apathy in those with SIVD [7], whereas others have reported a higher prevalence of sleep disturbances, appetite changes and aberrant motor behavior in those with AD [11]. Identifying differences between patients with SIVD and AD in relation to different stages of dementia is important, as prompt recognition of BPSD may mitigate severe consequences related to cognitive and functional deterioration [12].

Exploring the etiologies of BPSD can be challenging, as medications, environmental triggers, and unmet needs [13] are regarded to be contributing factors in addition to preexisting brain structural changes relevant to the subtype and severity of dementia [6, 7]. The corpus callosum (CC) has been associated with numerous neuropsychiatric features among demented patients, as it is involved in the control of mood [14], cognition [15, 16], and behavior [17]. It is the largest white matter structure in the human brain, and is comprised of widespread homotopic and heterotopic connections according to the anatomical portion. Fibers within the anterior portion of the CC, the genu (GCC), project mainly toward the prefrontal cortex [18]. The posterior portion of the CC, known as the splenium (SCC), communicates somatosensory information between the parietal and occipital lobes and extends the interconnections along the lateral surface of the occipital and temporal horns of the lateral ventricles [19]. In contrast to the GCC or SCC, the body of the corpus callosum (BCC) appears to be comprised of inter-hemispheric fibers and connections between anterior and posterior poles [18]. Disruption of each individual portion of the CC may have different effects on cognitive and psychiatric performance. Diffusion tensor imaging (DTI) is a useful tool to detect microstructural changes within white matter. Some studies have reported that changes in white matter integrity within the anterior cingulate are associated with apathy in patients with AD or mild cognitive impairment [20, 21]. However, some DTI studies also addressed the role of regions other than the CC to be responsible for apathy. In one DTI study using tract-based spatial statistics, the severity of apathy was negatively correlated with microstructural alterations of the uncinate fasciculus, cingulum, and superior longitudinal fasciculus in addition to the CC [17]. Another DTI study using a voxel-based approach also concluded apathy in AD is associated with impaired white matter integrity in the parietal regions and medial thalamus in addition to the anterior cingulate [22]. Some other study also reported significant associations between global changes of major white matter tracts with BPSD [23]. To date, few studies have investigated the relationship between BPSD and changes in DTI within the CC in patients with SIVD. We therefore used the Neuropsychiatric Inventory (NPI) and DTI in patients with SIVD and AD in the current study to (i) describe and compare BPSD in these patients according to different stages of disease and (ii) explore the association between the NPI parameters and DTI within the CC as well as other major fibers.

## Methods

Twenty-four community-living patients with SIVD and 32 with AD who visited the Department of Neurology of our hospital from July 2014 to June 2016 were consecutively recruited. The demographic data and results of serology tests, general cognitive function assessment, NPI, conventional brain magnetic resonance imaging (MRI), and DTI of each patient were recorded. This study was approved by the Institutional Review Board of our hospital (REC 103–14). All participants and their caregivers provided their written informed consent to participate in this study.

### Inclusion and exclusion criteria

Patients were defined as having SIVD if they had: (1) cognitive complaints that interfered with complex occupational and social activities [10]; (2) appearances of subcortical ischemic changes on brain MRI [10]; (3) Mini-Mental State Examination (MMSE) score ≤ 26 [24]; and (4) Hachinski Ischemic Scale ≥7 [25]. Patients were defined as having AD if they had: (1) cognitive concerns reflecting a change in cognition reported by the patient, informant or clinician [26]; (2) absence of profound subcortical ischemic changes on brain MRI [10]; (3) MMSE score ≤ 26 [24]; and (4) Hachinski Ischemic Scale score ≤ 4 [25].

The exclusion criteria were: (1) state of delirium or history of severe psychiatric disorders; (2) stroke event within the past 2 weeks; (3) appearance of cortical and/or cortico-subcortical non-lacunar territorial infarcts and watershed infarcts, hemorrhages, signs of normal pressure hydrocephalus, and specific causes of white matter lesions (e.g. multiple sclerosis, sarcoidosis, brain irradiation) [10]; (4) derangements in serology tests that may have contributed to cognitive impairment such as abnormal levels of free T4, cortisol, folic acid, vitamin B12, or rapid plasma reagin; and (5) severe hearing or visual impairment.

### Demographic data registry

The systemic diseases of the patients were recorded. Hypertension was defined as a systolic blood pressure ≥ 140 mmHg or a diastolic blood pressure ≥ 90 mmHg at two separate blood pressure measurements [27], self-report of a diagnosis of hypertension, or medical treatment. Diabetes mellitus was defined as a fasting blood sugar level ≥ 126 mg/dl, random postprandial blood sugar level ≥ 200 mg/dl, HbA1C ≥ 6.5% [28], self-report of a diagnosis of diabetes mellitus, or treatment with insulin or oral hypoglycemic agents. Chronic kidney disease was defined as a glomerular filtration rate according to the Modification of Diet in Renal Disease Study eq. [29] < 60 mL per minute per 1.73 m2 for ≥3 months with or without evidence of kidney damage [30]. Coronary artery disease was defined as an event and/or history related to stable angina pectoris, unstable angina pectoris, or myocardial infarction [31]. To avoid the confounding effect of medications on cognitive performance and neuropsychiatric symptoms, the current use (within 1 month) of antipsychotics, anxiolytics, and antidepressants was reviewed and recorded.

### Serology test

Antecubital venous blood samples were collected after an 8-h fast for hemogram, serum creatinine, folate, vitamin B12, free T4, thyroid stimulating hormones, cortisol, and rapid plasma reagin measurements. Samples were collected in evacuated tubes containing EDTA, centrifuged within 10 min and stored below −20 °C until analysis.

### General cognitive function assessment

General cognitive function assessment included Clinical Dementia Rating (CDR) [32], the Taiwanese version of the MMSE [24], and Cognitive Abilities Screening Instrument (CASI) [33]. The CASI is an objective test with nine cognitive domains including attention, mental manipulation, orientation, short-term memory, long-term memory, language, visual construction, category fluency, and abstraction and judgement [33]. The raw scores in our AD patients were transformed to standard score (z score) according to normative data in Taiwan, aiming on examining the cognitive profiles of the AD patients [32]. The CDR is a semi-structured interview with the patient and a reliable informant. It characterizes six domains of cognitive and functional performance including memory, orientation, judgment and problem solving, community affairs, home and hobbies, and personal care. An overall score is reached according to a standardized algorithm. A CDR score of 0 denotes no cognitive impairment, with the remaining four scores representing various stages of severity (0.5: very mild; 1: mild; 2: moderate; 3: severe) [32]. Both the MMSE and CASI assess global cognition of the subjects, with a higher score representing better cognition [24, 33].

### NPI

Caregivers (Spouse and/or children) were chosen as the source of information for this inventory based onto the reasons that patients with cognitive deficits may not explicitly recall their symptoms and demonstrate behavioral problems during the interview. Therefore, their caregivers were regarded to be optimal reporter to assist NPI assessment. The NPI items were further classified into four domains according to factor analysis previously reported by Aalten et al. [34]. These four domains and the included items were: (i) hyperactivity: including agitation, disinhibition, irritability, and aberrant motor behavior; (ii) psychosis: including delusions, hallucinations and night-time behavior; (iii) affective: including depression and anxiety; and (iv) apathy: including apathy and eating abnormalities. Both SIVD and AD groups were divided into very-mild dementia (CDR 0.5) and mild-to-moderate dementia (CDR 1 ~ 2) subgroups for comparisons of psychiatric domains.

### Conventional brain MRI

Brain MRI using a 3.0 T scanner (Discovery MR750, GE Medical Systems, Milwaukee, WI) was performed in all patients. White matter hyperintensities were rated in accordance to the scale proposed by Fazekas [35] from T2 fluid-attenuated inversion recovery (T2-FLAIR) sequences in the axial plane by a single rater (Min-Chien Tu). The parameters were as follows: repetition time 12,000 ms, echo time 120 ms, inversion time 2200 ms, slice thickness 5 mm, field of view 24 cm, and matrix 256 × 256. In the Fazekas scale, composite scores are derived from the summation of periventricular white matter hyperintensities (0 = absent; 1 = “caps” or pencil-thin lining; 2 = smooth “halo”; 3 = irregular periventricular signal extending into the deep white matter) and deep white matter hyperintensities (0 = absent; 1 = punctate foci; 2 = beginning confluence; 3 = large confluent areas).

### DTI

**Fig. 1:**
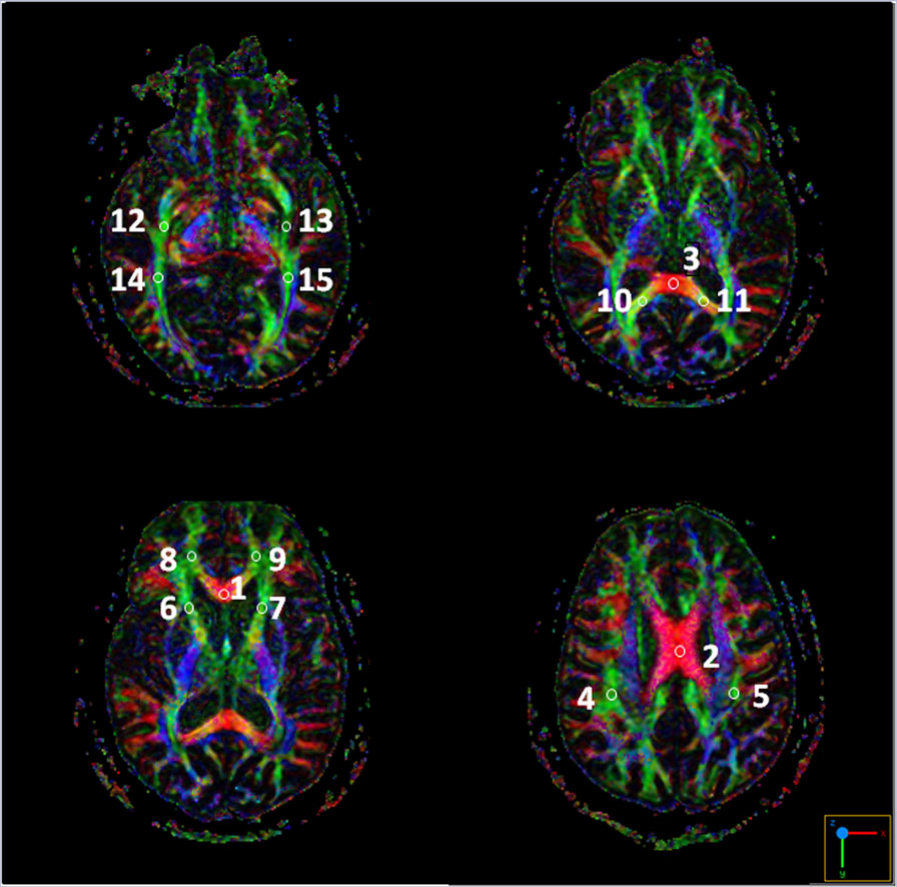
Template for regions of interest in diffusion tensor imaging parameters measurement (1: the genu of the corpus callosum; 2: the body of the corpus callosum; 3: the splenium of the corpus callosum; 4/5: the right/left superior longitudinal fasciculus; 6/7: the right/left anterior thalamic radiation; 8/9: the right/left forceps minor; 10/11: the right/left forceps major; 12/13:the right/left uncinate fasciculus; 14/15: the right/left inferior longitudinal fasciculus)

DTI data were acquired using a single-shot spin-echo echo-planar imaging sequence. The diffusion-sensitizing gradients were applied along 20 non-collinear directions with diffusion weighting factor b = 1000 s/mm^2^, plus one b = 0 image. The imaging parameters were: TR/TE = 8000/82 ms, matrix size = 128 × 128, field of view = 240 mm, slice thickness = 3 mm without intersection gap, number of excitations = 2, number of slices = 67, scan time = 5 min and 58 s. The post-processing software Functool (GE Medical System, Milwaukee, WI) was used to measure fractional anisotropy (FA) and mean diffusivity (MD) values in different slices of B0 and color-coded maps on the axial images. Each region of interest (ROI), presumed to be circular in shape with a consistent size (30–35 mm^2^) in all of the patients to maintain a stable number of voxels, was manually drawn by a single rater (Min-Chien Tu). One voxel size of the resulting DTI images was 1.875 mm × 1.875 mm × 3 mm. Each ROI therefore represented 9–10 voxels (size: 30–35 mm2, slide thickness: 3 mm). The positions of the ROIs were compared to the corresponding slices of the T2-FLAIR axial data sets to avoid measurements within regions of lacunes and white matter hyperintensities. The midline GCC, BCC, and SCC were selected to be ROIs for FA and MD measurements to investigate correlations with the psychiatric domains. In addition, ROIs including the superior longitudinal fasciculus, anterior thalamic radiation, forceps minor, forceps major, uncinate fasciculus, and inferior longitudinal fasciculus, were evaluated symmetrically within bilateral hemispheres. FA was calculated with values ranging from zero to one, where a higher value indicated a greater degree of WM integrity. In contrast, higher MD values indicated a greater degree of WM damage. The DTI parameters of each ROI were obtained from the averaged value of two adjacent slices. The template was shown in Fig. 1.

### Statistical analysis

The independent *t*-test and *x* ^*2*^ test were used to detect group differences in demographic, morphometric and psychometric data where appropriate. Partial correlation analysis was used to evaluate associations between NPI and DTI parameters, with the aim of controlling for confounding factors including age, the total score of CASI, and the CDR score. To determine the reliability of DTI measurements, the same rater repeated ROI selections within the CC on all subjects in this study. The intra-observer reliability was assessed using the averages of intra-class correlation coefficients with absolute agreement, and was calculated for all patients together for each portion of the CC. In current study, we used a “Two-Way Random” effects model with absolute agreement. This model fits the condition that the dependent variables (DTI parameters) are assessed by the same rater, where both an effect of rater and of ratee (i.e. two effects) is considered and both rater and ratee are drawn randomly from larger populations (i.e. a random effects model). The intra-class correlation coefficients values were considered to indicate excellent agreement and substantial agreement if they were greater than 0.8 [35]. In addition, to examine the effect of individual regional microstructural changes on severity of behavioral and psychological symptoms independent of whole-brain white matter damages, stepwise linear regressions between each domain of behavioral and psychological symptoms and DTI parameters of all ROIs were tested controlling for Fazekas scale and Hachinski Ischemic Scale, as well as group, the total score of the CASI, CDR, and age. All statistical tests were performed using SPSS software version 19 (IBM, Armonk, New York). A *p* value less than 0.05 was considered to be statistically significant.

## Results

### Sample characteristics

Table 1 shows comparisons of the demographic data and clinical characteristics between the SIVD and AD groups. There were no significant difference between the two groups in age (*t* = −0.67, *p* = 0.509), gender (*x* ^*2*^ = 0.29, *p* = 0.589), or education (*t* = 0.96, *p* = 0.342). The SIVD group had higher Fazekas scale (*t* = 8.00, *p* < 0.001) and Hachinski (*t* = 10.63, *p* < 0.001) scores than the AD group. There were no significant differences in MMSE (*t* = −1.49, *p* = 0.141), CASI (*t* = −1.52, *p* = 0.136), and CDR sum-of-box (*t* = 1.04, *p* = 0.304) scores between the two groups. There was also no significant difference in the severity of dementia between the two groups (*x* ^*2*^ = 1.82, *p* = 0.402). With regards to systemic diseases, the SIVD group had a higher rate of coronal artery disease (*x* ^*2*^ = 12.06, *p* = 0.001) and diabetes mellitus (*x* ^*2*^ = 7.67, *p* = 0.006). With regards to medications, the use of acetylcholinesterase inhibitors was significantly higher in the AD group (*x* ^*2*^ = 18.31, *p* < 0.001). There was no difference in the use of other psychotropic medications (*x* ^*2*^ = 0.01 ~ 3.23, *p* = 0.072 ~ 0.916). In AD patients, the mean CASI z scores of total scores, orientation, and short-term memory domains were the lowest and lower than the defective cutoff score of −1.5 in AD patients with a CDR score 1 ~ 2. The mean z score of short-term memory domain was also the lowest and lower than lower than −1.5 in patients with a CDR score 0.5 (Additional file 1).

**Table 1:** Basic information

	SIVD (*n* = 24)		AD (*n* = 32)		T-test	
	Mean	*SD*	Mean	*SD*	*t*	*p*
Age(years)	71.71	12.01	73.59	8.00	−0.67	0.509
Education(years)	5.96	3.69	5.00	3.71	0.96	0.342
Fazekas scale	4.67	1.09	2.13	1.24	8.00	< 0.001***
Hachinski score	7.63	2.92	1.09	0.86	10.63	< 0.001***
Mini-Mental State Examination	19.08	5.04	21.03	4.67	−1.49	0.141
Cognitive Abilities Screening Instrument	61.04	14.78	67.19	15.20	−1.52	0.136
CDR sum-of –box	4.48	3.86	3.53	2.58	1.04	0.304
				Chi-square		
	*n*	% of presence	*n*	% of presence	*x* ^*2*^	*p*
Gender (Male/Female)	13/11	54.2/45.8	15/17	46.9/53.1	0.29	0.589
Global CDR					1.82	0.402
CDR = 0.5	14	58.3	21	65.6		
CDR = 1	7	29.2	10	31.3		
CDR = 2	3	12.5	1	3.1		
System disease						
Hypertension	13	54.2	12	37.5	1.54	0.214
Diabetes mellitus	12	50.0	5	15.6	7.67	0.006**
Chronic kidney disease	7	29.2	4	12.5	2.41	0.120
Coronal artery disease	11	45.8	2	6.3	12.06	0.001**
Medication						
Acetylcholinesterase inhibitors	0	0.0	17	53.1	18.31	< 0.001***
Antidepressants	4	16.7	5	15.6	0.01	0.916
Antipsychotics	0	0.0	1	3.1	0.76	0.382
Benzodiazepines	0	0.0	4	12.5	3.23	0.072

*Abbreviation*: *SIVD* subcortical ischemic vascular disease, *AD* Alzheimer’s disease, *CDR* Clinical Dementia Rating, *SD* Standard deviation

**p < 0.01

***p < 0.001 on comparisons between SIVD and AD

### Cognitive status and neuropsychiatric symptoms among the patients with dementia.

Table 2 shows comparisons of cognitive status and the NPI in the patients with respect to the severity and subtypes of dementia. The patients with AD and SIVD had comparable cognitive status across different stages of dementia. With regard to those with a CDR score of 0.5, the total MMSE and CASI scores in the SIVD group were 21.07 ± 4.34 and 67.21 ± 13.16, respectively, compared to 22.90 ± 3.75 and 73.29 ± 13.12, respectively, in the AD group. With regards to those with a CDR score 1 ~ 2, the total MMSE and CASI scores in the SIVD group were 16.30 ± 4.79 and 52.40 ± 12.87, respectively, compared to 17.45 ± 4.25 and 55.55 ± 12.04, respectively, in the AD group. No in-group significant differences were noted (*p* = 0.189 ~ 0.570). The patients with AD and SIVD both presented a lower cognitive score in those with CDR score = 1 ~ 2 than those with CDR score = 0.5 (*p* < 0.001 ~ 0.018). Overall, the patients with a CDR score 1 ~ 2 had higher scores in the symptoms of delusion (*t* = 2.33, *p* = 0.030), apathy (*t* = 2.83, *p* = 0.009), and the apathy domain (*t* = 2.40, *p* = 0.022) than the patients with a CDR score 0.5. Similarly, scores in the symptom of apathy (*t* = 4.51, *p* = 0.001) and apathy domain (*t* = 2.99, *p* = 0.007) were significantly higher in the patients with a CDR score of 1 ~ 2 than those with a CDR score of 0.5 in the SIVD group. There were no significant differences in the different stages of severity in the AD group (*t* = −1.94 ~ 2.01, *p* = 0.063 ~ 0.869). Comparing NPI profiles between the patients with AD and SIVD across all stages, the patients with SIVD had a higher score in the symptom of apathy (*t* = 2.27, *p* = 0.031). To clarify the impact of the severity of dementia, group comparisons confined within individual stages were performed. In the patients with a CDR score of 1 ~ 2, those with SIVD had significantly higher scores in both apathy domain (*t* = 3.21, *p* = 0.005) and the symptom of apathy (*t* = 4.18, *p* = 0.004) than the patients with AD. In contrast, there were no significant differences in neuropsychiatric symptoms between the two groups during the stage of a CDR score of 0.5 (*t* = −1.25 ~ 1.71, *p* = 0.189 ~ 0.868).

**Table 2:** Cognitive status and Neuropsychiatric Inventory among dementia patients

	SIVD			AD		
	All (*n* = 24)	CDR = 0.5 (*n* = 14)	CDR =1 ~ 2 (*n* = 10)	All (*n* = 32)	CDR = 0.5 (*n* = 21)	CDR = 1 ~ 2 (*n* = 11)
MMSE^£££ ǂ $$$^	19.08 ± 5.04	21.07 ± 4.34	16.30 ± 4.79	21.03 ± 4.67	22.90 ± 3.75	17.45 ± 4.25
CASI^£££ ǂ $$$^	61.04 ± 14.78	67.21 ± 13.16	52.40 ± 12.87	67.19 ± 15.20	73.29 ± 13.12	55.55 ± 12.04
Total score	6.42 ± 5.72	5.71 ± 6.33	7.40 ± 4.88	6.88 ± 8.87	5.19 ± 5.39	10.09 ± 12.98
Hyperactivity	0.51 ± 0.82	0.48 ± 0.94	0.55 ± 0.67	0.45 ± 0.89	0.18 ± 0.29	0.98 ± 1.35
Agitation	0.50 ± 1.45	0.64 ± 1.74	0.30 ± 0.95	0.38 ± 1.19	0.00 ± 0.00	1.09 ± 1.87
Disinhibition	0.00 ± 0.00	0.00 ± 0.00	0.00 ± 0.00	0.19 ± 1.06	0.00 ± 0.00	0.55 ± 1.81
Irritability	1.21 ± 1.82	1.29 ± 2.16	1.10 ± 1.29	0.91 ± 1.59	0.71 ± 1.15	1.27 ± 2.24
Aberrant motor behavior	0.33 ± 1.63	0.00 ± 0.00	0.80 ± 2.53	0.34 ± 1.15	0.00 ± 0.00	1.00 ± 1.84
Psychosis	0.58 ± 0.78	0.57 ± 0.86	0.60 ± 0.68	0.79 ± 1.19	0.65 ± 0.75	1.06 ± 1.78
Delusion^£^	0.21 ± 0.72	0.00 ± 0.00	0.50 ± 1.08	0.63 ± 1.91	0.00 ± 0.00	1.82 ± 2.99
Hallucination	0.08 ± 0.41	0.00 ± 0.00	0.20 ± 0.63	0.31 ± 1.15	0.10 ± 0.44	0.73 ± 1.85
Night-time behavior	1.46 ± 2.27	1.71 ± 2.59	1.10 ± 1.79	1.44 ± 1.97	1.86 ± 2.10	0.64 ± 1.43
Affective	0.63 ± 1.10	0.79 ± 1.25	0.40 ± 0.84	1.08 ± 1.41	1.05 ± 1.40	1.14 ± 1.50
Depression	0.58 ± 1.25	0.57 ± 1.22	0.60 ± 1.35	0.88 ± 1.50	0.76 ± 1.14	1.09 ± 2.07
Anxiety	0.67 ± 1.52	1.00 ± 1.88	0.20 ± 0.63	1.28 ± 2.33	1.33 ± 2.20	1.18 ± 2.68
Apathy ^£ǂǂ^	0.65 ± 0.89	0.25 ± 0.80	1.20 ± 0.72^§§^	0.23 ± 0.51	0.21 ± 0.46	0.27 ± 0.61^§§^
Apathy ^££ǂǂ^	0.96 ± 1.43^*^	0.07 ± 0.27	2.20 ± 1.48^§§^	0.25 ± 0.62^*^	0.29 ± 0.72	0.18 ± 0.41^§§^
Eating abnormalities	0.33 ± 1.27	0.43 ± 1.60	0.20 ± 0.63	0.22 ± 0.87	0.14 ± 0.66	0.36 ± 1.21

(1) Mean ± *SD* were shown. (2) *Abbreviation*: *SIVD* subcortical ischemic vascular disease, *AD* Alzheimer’s disease, *CDR* Clinical Dementia Rating, *MMSE* Mini-Mental State Examination, *CASI* Cognitive Abilities Screening Instrument, *Sig.* Significance.(3) T-test was used to evaluate the group difference and significant difference was defined as *p* value <0.05. (4) ^*^ *p* < 0.05 on the comparisons between all SIVD patients and all AD patients;^#^ *p* < 0.05 on the comparisons between SIVD patients with CDR score = 0.5 and AD patients with CDR score = 0.5; ^§^ *p* < 0.05, ^§§^ *p* < 0.01 on the comparisons between SIVD patients with CDR score = 1 ~ 2 and AD patients with CDR score = 1 ~ 2. (5) ^£^ *p* < 0.05, ^££^ *p* < 0.01, and ^£££^ *p* < 0.001 on the comparisons between patient with CDR score = 0.5 and with CDR score = 1 ~ 2 among all patients respectively. (6) ^ǂ^ *p* < 0.05 and ^ǂǂ^ *p* < 0.01 on the comparisons between patient with CDR score = 0.5 and with CDR score = 1 ~ 2 in SIVD group respectively. (7) ^$$$^ *p* < .001 on the comparisons between patient with CDR score = 0.5 and with CDR score = 1 ~ 2 in AD group

### Repeatability and variation of the DTI measurements

The intra-class correlation coefficients ranged from 0.95 to 0.97 in FA and 0.94 to 0.97 in MD values. The average intra-class correlation coefficients for FA was 0.956 and for ADC 0.956 (Additional file 2). There were lower levels of within-subject variability relative to between-subject variability.

### Correlations between NPI scores and DTI parameters within the CC

Table 3 shows correlations between the scores of NPI domains and the values of DTI within the CC in both patient groups. Overall, FA values in the SCC and MD values both in the BCC and SCC were significantly correlated with total NPI score (*r*_*FA splenium*_ = −0.27, *p*_*FA splenium*_ = 0.044; *r*_*MD body*_ = 0.32, *p*_*MD body*_ = 0.016; *r*_*MD splenium*_ = 0.38, *p*_*MD splenium*_ = 0.004;) and the domain of psychosis (*r*_*FA splenium*_ = −0.34, *p*_*FA splenium*_ = 0.010; *r*_*MD body*_ = 0.27, *p*_*MD body*_ = 0.041; *r*_*MD splenium*_ = 0.42, *p*_*MD splenium*_ = 0.001). MD values both in the BCC and SCC were also correlated with the hyperactivity (*r*_*body*_ = 0.29, *p*_*body*_ = 0.034; *r*_*splenium*_ = 0.27, *p*_*splenium*_ = 0.042) and apathy domains (*r*_*body*_ = 0.29, *p*_*body*_ = 0.031; *r*_*splenium*_ = 0.27, *p*_*splenium*_ = 0.044). On controlling for age, the total score of the CASI, and CDR, MD values in the SCC remained to be positively correlated with the total NPI score (*p* = 0.047) and the psychosis domain (*p* = 0.010).

**Table 3:** Correlation between Neuropsychiatric Inventory and diffusion tensor imaging parameters within corpus callosum across patient groups

		Fractional Anisotropy			Mean Diffusivity		
	Neuropsychiatric Inventory	Genu	Body	Splenium	Genu	Body	Splenium
Total patients (*n* = 56)	Total score	0.05	−0.20	−0.27*	−0.06	0.32*	0.38^**ǂ^
	Hyperactivity	−0.01	−0.20	−0.19	−0.01	0.29*	0.27^*^
	Psychosis	0.02	−0.16	−0.34*	0.01	0.27*	0.42^**ǂ^
	Affective	0.23	0.06	0.04	−0.20	0.04	0.06
	Apathy	−0.10	−0.26	−0.25	0.04	0.29*	0.27^*^
SIVD patients (*n* = 24)	Total score	0.12	−0.36	−0.40	−0.30	0.37	0.43^*^
	Hyperactivity	0.03	−0.31	−0.16	−0.20	0.28	0.19
	Psychosis	0.03	−0.32	−0.49^*ǂǂ^	−0.10	0.27	0.42^*ǂ^
	Affective	0.33	0.07	0.03	−0.31	0.03	0.06
	Apathy	−0.02	−0.14	−0.32	−0.14	0.17	0.32
AD patients (*n* = 32)	Total score	0.01	−0.16	−0.24	0.07	0.46**	0.40^*ǂ^
	Hyperactivity	−0.02	−0.08	−0.21	0.09	0.36*	0.33
	Psychosis	−0.05	−0.17	−0.34	0.11	0.50**	0.49^**ǂ^
	Affective	0.08	−0.10	−0.02	−0.06	0.24	0.14
	Apathy	0.05	−0.24	−0.02	0.01	0.29	0.11

(1) Pearson’s correlation coefficient was used. (2) *p < 0.05, **p < 0.01 on the correlation between NPI and DTI parameters. (3) ^ǂ^ *p* < 0.05, ^ǂǂ^ *p* < 0.01 on the correlation between NPI and DTI parameters on controlling of age, the total score of CASI, and the CDR score

In the SIVD group, the MD value in the SCC was correlated with total NPI score (*r* = 0.43, *p* = 0.036). Both FA (*r* = −0.49, *p* = 0.016) and MD (*r* = 0.42, *p* = 0.039) values in the SCC were correlated with the psychosis domain; the significant associations remained after controlling for age, the total score of the CASI, and CDR (*p* = 0.009 ~ 0.020).

Similar to the correlations in the SIVD group, MD values in the SCC but also in the BCC were correlated with total NPI score (*r*_*MD body*_ = 0.46, *p*_*MD body*_ = 0.009; *r*_*MD splenium*_ = 0.40, *p*_*MD splenium*_ = 0.024) and the psychosis domain (*r*_*MD body*_ = 0.50, *p*_*MD body*_ = 0.004; *r*_*MD splenium*_ = 0.49, *p*_*MD splenium*_ = 0.005) in the AD group. The MD value in the BCC was also correlated with the hyperactivity domain (*r* = 0.36, *p* = 0.044). Consistent with the aforementioned analysis of overall patients, MD values in the SCC was positively correlated with the total NPI score and the psychosis domain (*p* = 0.017 ~ 0.024) on controlling for age, the total score of the CASI, and CDR.

### Regression models for NPI domains and whole-brain DTI parameters

To examine the effect of regional microstructural changes on severity of behavioral and psychological symptoms independent of whole-brain white matter damages, stepwise linear regressions were tested (Table 4). Each domain of behavioral and psychological symptoms was taken as the dependent variable, and DTI parameters of all ROIs, the Fazekas scale, Hachinski Ischemic Scale, group, the total score of the CASI, CDR, and age were taken as the independent variable. In this model, the Fazekas scale and Hachinski Ischemic Scale were taken to be the indices representing whole brain white matter damages.

**Table 4:** Regression models for Neuropsychiatric Inventory domains among patient groups

	Clinical factors					Fractional Anisotropy															Mean Diffusivity															
	Age	CASI	CDR	HIS	Fazekas scale	GCC	BCC	SCC	RSLF	LSLF	Rfminor	Lfminor	Rfmajor	Lfmajor	Ratr	Latr	Runc	Lunc	RILF	LILF	GCC	BCC	SCC	RSLF	LSLF	Rfminor	Lfminor	Rfmajor	Lfmajor	Ratr	Latr	Runc	Lunc	RILF	LILF	R^2^
All patients (*n* = 56)																																				
Hyperactivity	NA	NA	NA	NA	NA	NA	NA	NA	NA	−.320	NA	NA	NA	NA	NA	NA	NA	NA	NA	NA	NA	NA	NA	NA	NA	NA	NA	NA	NA	NA	NA	NA	NA	NA	NA	.102
Psychosis	NA	NA	NA	NA	NA	.316	NA	NA	NA	NA	NA	NA	NA	.296	NA	NA	NA	NA	NA	NA	NA	NA	**.** ***524***	NA	NA	NA	NA	NA	NA	NA	NA	NA	.312	NA	NA	.390
Affective	NA	NA	NA	NA	NA	NA	NA	NA	NA	NA	NA	NA	NA	NA	NA	NA	NA	NA	NA	NA	NA	NA	NA	NA	NA	NA	NA	NA	NA	NA	NA	NA	NA	NA	NA	NA
Apathy	NA	NA	.267	NA	NA	NA	NA	NA	NA	NA	NA	NA	NA	NA	NA	NA	NA	NA	NA	NA	NA	NA	NA	**.** ***436***	NA	NA	NA	NA	NA	NA	NA	NA	NA	NA	NA	.294
SIVD group (*n* = 24)																																				
Hyperactivity	NA	NA	NA	NA	NA	NA	NA	NA	NA	NA	NA	NA	NA	NA	NA	NA	NA	NA	NA	NA	NA	NA	NA	NA	**.** ***651***	NA	NA	NA	NA	NA	NA	NA	NA	NA	NA	.424
Psychosis	NA	NA	NA	NA	NA	NA	NA	NA	NA	NA	NA	NA	NA	NA	NA	NA	NA	NA	NA	NA	NA	NA	NA	NA	NA	NA	NA	NA	NA	NA	NA	NA	.490	NA	NA	.240
Affective	NA	NA	NA	.347	NA	NA	NA	NA	NA	NA	NA	NA	**.** ***384***	NA	NA	NA	***−.806***	NA	NA	NA	**−.** ***684***	NA	NA	NA	NA	NA	NA	NA	NA	NA	NA	NA	NA	NA	NA	.721
Apathy	NA	NA	**.** ***546***	NA	NA	NA	NA	NA	***−.454***	NA	NA	**.** ***357***	NA	NA	NA	NA	NA	NA	***−.473***	NA	NA	NA	NA	NA	NA	**.** ***309***	NA	NA	NA	NA	NA	NA	NA	NA	NA	.815
AD group (*n* = 32)																																				
Hyperactivity	NA	NA	**.** ***546***	NA	NA	NA	NA	NA	NA	NA	NA	NA	NA	NA	NA	NA	NA	NA	NA	NA	NA	NA	NA	NA	NA	NA	NA	NA	NA	NA	NA\	NA	NA\	NA	**−.** ***475***	.401
Psychosis	NA	NA	NA	NA	NA	NA	NA	NA	NA	NA	NA	NA	NA	**.** ***539***	NA	NA	NA	NA	NA	NA	NA	.369	NA	NA	NA	NA	**.** ***450***	NA	NA	NA	−.300	NA	NA	NA	NA	.585
Affective	NA	NA	NA	NA	NA	NA	NA	NA	NA	NA	NA	NA	NA	NA	NA	NA	NA	NA	.415	NA	NA	NA	NA	NA	NA	NA	NA	NA	NA	NA	NA	NA	NA	NA	NA	.172
Apathy	NA	NA	NA	NA	NA	NA	NA	NA	NA	NA	NA	NA	NA	NA	NA	NA	NA	NA	NA	NA	NA	NA	NA	NA	**.** ***507***	NA	NA	NA	NA	NA	NA	NA	NA	NA	NA	.257

(1) The beta coefficients were noted. (2) The *p* value of all noted beta coefficients were less than 0.05, while the bold-faced italic values were less than 0.01. (3) *Abbreviation*: *NA* not available. *CASI* Cognitive Abilities Screening Instrument, *CDR* Clinical Dementia Rating, *HIS* Hachinski Ischemic Scale. *GCC* genu of the corpus callosum, *BCC* body of the corpus callosum, *SCC* splenium of the corpus callosum, *RSLF* right superior longitudinal fasciculus, *LSLF* left superior longitudinal fasciculus, *Rfminor* right forceps minor, *Lfminor* left forceps minor, *Rfmajor* right forceps major, *Lfmajor* left forceps major, *Ratr* right anterior thalamic radiation, *Latr* left anterior thalamic radiation, *Runc* right uncinate fasciculus, *Lunc* left uncinate fasciculus, *RILF* right inferior longitudinal fasciculus, and *LILF* left inferior longitudinal fasciculus

Regarding hyperactivity domain, the analysis found that FA value within the left superior longitudinal fasciculus (FA _*LSLF*_) (*β* = − 0.320, *t* = −2.478, *p* = 0.016) significantly predicted its severity (R^2^ = 0.102, F _(1,54)_ = 6.14, *p* = 0.016). Overall, the model accounted for 10% of the variance. The regression model was listed as below.

$$ \mathrm{Hyperactivity}\ \mathrm{domain}=1.612-2.731{\ FA}_{LSLF} $$

Regarding psychosis domain, the analysis found that MD value within the SCC (MD _*SCC*_) (*β* = 0.524, *t* = 4.073, *p* < 0.001), FA value within the left forceps major (FA _*Lfmajor*_) (*β* = 0.296, *t* = 2.552, *p* = 0.014), MD value within the left uncinate fasciculus (MD _*Lunc*_) (*β* = 0.312, *t* = 2.614, *p* = 0.012) and FA value within the GCC (FA _*GCC*_) (*β* = 0.316, *t* = 2.310, *p* = 0.025) significantly predicted its severity (R^2^ = 0.390, F_(4,51)_ = 8.16, *p* < 0.001). Overall, the model accounted for 39% of the variance.

MD _*SCC*_, FA _*Lfmajor*_, and MD _*Lunc*_ accounted for most of the variance (approximately 33%). The regression model was listed as below.

$$ \mathrm{Psychosis}\ \mathrm{domain}=-6.882+0.152{\ MD}_{SCC}+3.405{\ FA}_{Lfmajor} + 0.327{\ MD}_{Lunc}+2.331{\ FA}_{GCC} $$

Regarding apathy domain, the analysis found that MD value within the right superior longitudinal fasciculus (MD _*RSLF*_) (*β* = 0.436, *t* = 3.737, *p* < 0.001) and CDR (*β* = 0.267, *t* = 2.294, *p* = 0.026) significantly predicted its severity (R^2^ = 0.294, F_(2,53)_ = 11.06, *p* < 0.001). Overall, the model accounted for 29% of the variance. The regression model was listed as below.

$$ \mathrm{Apathy}\ \mathrm{domain}=-1.448+0.197{\ MD}_{RSLF} + 0.394\ CDR $$

No significant model regarding affective domain was identified.

To examine whether different factors existed in predicting individual NPI domains between patients with SIVD and AD, same stepwise regression analysis was performed. In SIVD patient, the most robust model is the one in predicting apathy domain, accounting for 82% of the variance. This model included CDR, FA values of the right superior longitudinal fasciculus/left forceps minor/right inferior longitudinal fasciculus, and MD value of the right forceps minor. In AD patient, the most robust model is the one in predicting psychosis domain, accounting for 59% of the variance. This model included FA value of the left forceps major, MD values of the BCC/left forceps minor/left anterior thalamic radiation. There was no overlapping between SIVD and AD patients regarding to factors predicting each individual NPI domains.

## Discussion

In this study, we compared the presentations of BPSD among patients with SIVD and AD at different stages of dementia. Using standardized neuropsychiatric instruments and DTI, distinct patterns of correlations and predictive factors between these two dementia subtypes were noted, thus providing further insight into the neurobiological mechanisms related to BPSD.

The results of previous studies on associations between neuropsychiatric manifestations and the severity of dementia in patients with AD and VaD have been inconsistent [4, 36, 37]. This may have been due to by different diagnostic criteria and neuroimaging tools. In one cohort study which recruited 28 pairs of patients with VaD and AD matched by demographic and cognitive profiles, the patients with VaD had more severe behavioral retardation, depression, and anxiety than those with AD [36]. A subsequent, larger study also reported a similar observation, where patients with VaD were prone to show more pronounced presentations of BPSD [37]. However, several studies have suggested that BPSD profiles between patients with VaD and AD may be different [4] or that they may vary according to VaD subgroup classification [7]. For example, a previous study using computerized tomography as the classification method indicated that patients with cortical, rather than subcortical VaD had a higher composite score of the apathy domain than those with AD [7]. Although this is quite different from our MRI-based study results, both studies highlight that cortico-subcortical circuits, and especially those involving frontal regions, are important neuronal substrates mediating initiation and motivation behavior [38, 39].

An apathy syndrome is defined as a syndrome of primary motivational loss [40]. Although such loss of motivation is traditionally regarded as not being attributable to emotional distress and/or intellectual impairment [40], recent studies have identified a strong association between apathy and lacunar volume in the white matter [41], as well as cognitive status [42, 43]. Our findings showed that the patients with SIVD presented with a higher severity of apathy than those with AD, which tended to be more pronounced with the severity of dementia. Apathy is frequently overlooked due to its negative symptom property, and it is often reported after profound cognitive decline and the development of other BPSD. Interestingly, our results indicated that the symptoms of apathy and depression evolved in a dissociative manner with the severity of dementia. Moreover, the apathy but not affective domain was significantly correlated with DTI parameters within the CC. Although psychomotor retardation and markedly diminished interest in daily activities may partially account for the clinical correlation between apathy and major depression, their underlying pathogenesis may not be completely identical [42]. The possibility of independent existence and/or different neuronal correlates underlying apathy and depression has been proposed in patients with stroke [42], AD [17], and small vessel disease [44]. Such findings suggest a unique pathogenesis related to apathy. In addition, we also found a significantly higher score of delusion in the later stages of dementia in the patients overall, which is consistent with previous studies [7, 45].

We also found an association between the severity of apathy and MD values within the SCC/BCC. Moreover, microstructural changes of the BCC/SCC appeared to correlate with the severity of apathy through the involvement of general cognition and dementia severity. Only a few studies have investigated the relationship between DTI changes and apathy among patients with dementia, and the results have been inconclusive [17, 21]. Several DTI studies have associated the severity of apathy with changes in FA within the left cingulum, right superior longitudinal fasciculus, GCC, BCC, SCC, and bilateral uncinate fasciculus [17, 21]. Another large cohort study reported that both median FA and MD values showed a widespread significant association with the severity of apathy among patients with small vessel disease [44]. In this study, we compared the impact of both MD and FA values within the CC on the severity of apathy, and found that the MD values were of greater clinical significance. Previous studies have suggested that FA reflects directional dependence of selected fiber tracts, and it is sensitive to changes in axonal membranes [46] and myelin integrity [47]. In contrast, MD represents a condition with a decrease in membrane or other barriers to free water diffusion [48]. We therefore hypothesize that compromised axon breakdown into free lipids and neutral fat in association with a generalized increase in extracellular space, as expected in the downstream process of Wallerian degeneration, contributes to the principle pathogenesis of apathy formation in both patients with AD and SIVD [49, 50]. Additionally, the remarkable correlation between apathy and microstructural changes within the BCC and SCC appeared to be modified by general cognition. This indicated the possibility that damages within the CC signify a widespread brain network derangement and relevant general cognitive decline. Our regression model also suggested the roles of the right superior longitudinal fasciculus, a region beyond the CC, and dementia severity in predicting the severity of apathy. The superior longitudinal fasciculus is an association fiber tract that is composed of several separate components. Due to its complex connections between variable prefrontal and parietal regions including angular gyrus, it is regarded to be critical for the perception process of visual space. The bidirectional fiber properties also constitute parts of attention network in regulation of spatial stimulus. Damages within the superior longitudinal fasciculus would therefore lead to attention deficits, which may aggravate clinical severity of apathy [51]. Our study results, especially those from SIVD patients, were also compatible with previous literatures, in which variable structures within the right hemisphere were regarded to be neural correlates of apathy [52, 53]. Therefore, DTI changes within the BCC and SCC may represent a surrogate marker for overall white matter integrity and general cognition based on their diverse fiber projections [18]. Taken together, the distinct association between the severity of apathy and integrity of the BCC/SCC may suggest that widespread white matter disconnection and defective cognition are the mechanisms of apathy.

Furthermore, microstructural changes within the SCC and BCC have been associated with psychosis through current DTI measures, and the clinical impact of changes in the SCC appeared to be more profound and across dementia subtypes. Mutism, hallucinations, psychosis, and hemispheric disconnections have been associated with a compromised splenium [54]. Disrupted interhemispheric communication and disintegration of misinterpreted stimulus have been proposed to be the possible pathogenesis [54]. Several cohort studies aimed at specific disease entities have also reported a similar phenomenon. A longitudinal study of patients with childhood-onset schizophrenia suggested that a failure of normal callosal growth may result in area reductions, particularly in the splenium, by early adulthood [55]. Another DTI study reported significant reductions in mean generalized FA values along the BCC and SCC in patients with bipolar 1 disorder, and that the FA values along the BCC were even lower among those with a history of psychotic features [56]. The authors therefore concluded that interhemispheric disconnectivity may be the key pathophysiological mechanism of psychosis [56]. However, another study reported that fibers of a small diameter from the heteromodal cortex which traverse the SCC and govern connections to temporo-occipital regions may also be more vulnerable to damage than fibers of a larger diameter connecting the unimodal motor and sensory cortex to the body [19]. It is therefore reasonable to infer that damage along the SCC would result in psychotic features and a secondary response of hyperactivity, as crosstalk between temporal and occipital regions is critical for integration of memory, identification, and visual stimulus perception [57]. Interestingly, our current research suggested that fibers other than the CC but under microstructural alterations in predicting psychosis were all confined within the left hemisphere, either in view of overall or AD patients Consistently, DTI changes within left hemisphere were reported among young people with psychosis [58], and even correlated with positive symptoms in drug-naïve schizophrenia patients [59]. There might be concern related to variable direction of linearity of each variable in predicting the psychosis domain. This reflects the fact that essential property of psychosis includes both positive and negative symptoms, and both of which could co-exist and interact in the same subject. Although studies using different neuroimaging tools [60, 61] have reported inconsistent findings related to the pathogenesis of psychosis, we were convinced that our DTI observation provided an informative spatial association between microstructural damages secondary to vascular burdens and psychosis formation.

The major strengths of this study include the recruitment of a representative hospital-based sample of elderly patients under a standardized DTI protocol and neuropsychiatric assessments. In addition, the complex relationships among brain structure, cognition, and psychiatric symptoms were thoroughly investigated. A potential limitation of this study is the reliance on clinical, rather than pathological, diagnoses. Biomarkers related to the pathology of AD such as cerebrospinal fluid tau protein and Abeta42 could be incorporated into future studies. A longitudinal study design, especially for those with a CDR score of 0.5, would also further confirm the accuracy of the clinical diagnoses. However, it is worthy to point out that we had examined the cognitive profiles in our AD patients, which appeared to be categorized as the ones with amnestic presentations. Second, the DTI changes within the CC may reveal an epiphenomenon of dementia progression and/or a state of global white matter damages, rather than a direct reflection of neural substrates of BPSD. We therefore carefully examine the effect of the whole-brain vascular burden and microstructural changes within major fibers on BPSD, and highlight several regions of clinical significance regarding specific domains of BPSD formation and dementia subtypes. Third, as the present study highlights apathy and psychosis from clinical and/or neuroimaging perspectives, integrating tools detailing the severity and impact of apathy (e.g., Apathy Evaluation Scale) and psychosis symptoms (e.g., Psychotic Symptom Rating Scales) in future studies would provide more information related to psychological biometrics.

## Conclusions

In summary, we found that both apathy and delusion were more profound in line with the severity of dementia than other psychiatric symptoms, especially in the patients with SIVD. In addition, apathy appeared to be more profound in the patients with SIVD than in those with AD. Furthermore, symptoms of apathy and depression evolved in a dissociative manner with the severity of dementia, implying the possibility of different underlying neuronal correlates. White matter integrity within the BCC and SCC was correlated with the domains of hyperactivity, psychosis, and apathy, as well as the total score of NPI. Additionally, our study also identified important roles of regions other than the CC to individual domain of BPSD, including the left superior longitudinal fasciculus to the hyperactivity domain, the left uncinate fasciculus/forceps major to the psychosis domain, and the right superior longitudinal fasciculus to the apathy domain. Contrasting the relationship between the psychosis domain with microstructural alterations within the left hemisphere, apathy among the patients with dementia was associated with general cognition, dementia severity, and a state of fiber disconnection within the right hemisphere. A discernible pattern of clinical and DTI factors in predicting BPSD existed between SIVD and AD patients.

## Additional files

Additional file 1: Cognitive function in standard scores (z score) among patient with Alzheimer’s disease. (DOC 47 kb)

Additional file 2: Intra-class correlation coefficients of measurement of diffusion tensor imaging parameters (0 ≤ fractional anisotropy ≤1; mean diffusivity: in units of m^2^ s^−1^ × 10^−9^). (DOC 45 kb)

## Abbreviations

AD: Alzheimer’s disease
BCC: the body of the corpus callosum
BPSD: behavioral and psychological symptoms of dementia
CASI: Cognitive Abilities Screening Instrument
CC: the corpus callosum
CDR: Clinical Dementia Rating
DTI: diffusion tensor imaging
FA: fractional anisotropy
MD: mean diffusivity
MMSE: Mini-Mental State Examination
NPI: Neuropsychiatric Inventory
ROI: region of interest
SCC: the splenium of the corpus callosum
SIVD: subcortical ischemic vascular disease
T2-FLAIR: T2 fluid-attenuated inversion recovery
VaD: vascular dementia
