# Correction to: Comparison of neuropsychiatric symptoms and diffusion tensor imaging correlates among patients with subcortical ischemic vascular disease and Alzheimer’s disease

**DOI:** 10.1186/s12883-017-0969-0

**Published:** 2017-10-02

**Authors:** Min-Chien Tu, Wen-Hui Huang, Yen-Hsuan Hsu, Chung-Ping Lo, Jie Fu Deng, Ching-Feng Huang

**Affiliations:** 1 Department of Neurology, Taichung Tzu Chi Hospital, Buddhist Tzu Chi Medical Foundation, No. 88, Sec. 1, Fengxing Rd., Tanzi Dist, 427 Taichung City, Taiwan; 2 School of Medicine, Tzu Chi University, Hualien, Taiwan; 3 Department of Psychology, National Chung Cheng University, Chiayi, Taiwan; 4 Department of Radiology, Taichung Tzu Chi Hospital, Buddhist Tzu Chi Medical Foundation, Taichung, Taiwan

After publication of this article [1] it was brought to our attention that there was an error in the alignment of rows in Table 4. As such the corrected version of Table 4 is shown below, with the alignment corrected.

**Table 4:** Regression models for Neuropsychiatric Inventory domains among patient groups

	Clinical factors					Fractional Anisotropy															Mean Diffusivity															
	age	CASI	CDR	HIS	Fazekas scale scale	GCC	BCC	SCC	RSLF	LSLF	Rfminor	Lfminor	Rfmajor	Lfmajor	Ratr	Latr	Runc	Lunc	RILF	LILF	GCC	BCC	SCC	RSLF	LSLF	Rfminor	Lfminor	Rfmajor	Lfmajor	Ratr	Latr	Runc	Lunc	RILF	LILF	R^2^
**All patients (** ***n*** **= 56)**																																				
Hyperactivity	NA	NA	NA	NA	NA	NA	NA	NA	NA	−.320	NA	NA	NA	NA	NA	NA	NA	NA	NA	NA	NA	NA	NA	NA	NA	NA	NA	NA	NA	NA	NA	NA	NA	NA	NA	.102
Psychosis	NA	NA	NA	NA	NA	.316	NA	NA	NA	NA	NA	NA	NA	.296	NA	NA	NA	NA	NA	NA	NA	NA	**.** ***524***	NA	NA	NA	NA	NA	NA	NA	NA	NA	.312	NA	NA	.390
Affective	NA	NA	NA	NA	NA	NA	NA	NA	NA	NA	NA	NA	NA	NA	NA	NA	NA	NA	NA	NA	NA	NA	NA	NA	NA	NA	NA	NA	NA	NA	NA	NA	NA	NA	NA	NA
Apathy	NA	NA	.267	NA	NA	NA	NA	NA	NA	NA	NA	NA	NA	NA	NA	NA	NA	NA	NA	NA	NA	NA	NA	**.** ***436***	NA	NA	NA	NA	NA	NA	NA	NA	NA	NA	NA	.294
**SIVD group (** ***n*** **= 24)**																																				
Hyperactivity	NA	NA	NA	NA	NA	NA	NA	NA	NA	NA	NA	NA	NA	NA	NA	NA	NA	NA	NA	NA	NA	NA	NA	NA	**.** ***651***	NA	NA	NA	NA	NA	NA	NA	NA	NA	NA	.424
Psychosis	NA	NA	NA	NA	NA	NA	NA	NA	NA	NA	NA	NA	NA	NA	NA	NA	NA	NA	NA	NA	NA	NA	NA	NA	NA	NA	NA	NA	NA	NA	NA	NA	.490	NA	NA	.240
Affective	NA	NA	NA	.347	NA	NA	NA	NA	NA	NA	NA	NA	**.** ***384***	NA	NA	NA	***−.806***	NA	NA	NA	**−.** ***684***	NA	NA	NA	NA	NA	NA	NA	NA	NA	NA	NA	NA	NA	NA	.721
Apathy	NA	NA	**.** ***546***	NA	NA	NA	NA	NA	***−.454***	NA	NA	**.** ***357***	NA	NA	NA	NA	NA	NA	***−.473***	NA	NA	NA	NA	NA	NA	**.** ***309***	NA	NA	NA	NA	NA	NA	NA	NA	NA	.815
**AD group (** ***n*** **= 32)**																																				
Hyperactivity	NA	NA	**.** ***546***	NA	NA	NA	NA	NA	NA	NA	NA	NA	NA	NA	NA	NA	NA	NA	NA	NA	NA	NA	NA	NA	NA	NA	NA	NA	NA	NA	NA	NA	NA	NA	**−.** ***475***	.401
Psychosis	NA	NA	NA	NA	NA	NA	NA	NA	NA	NA	NA	NA	NA	**.** ***539***	NA	NA	NA	NA	NA	NA	NA	.369	NA	NA	NA	NA	**.** ***450***	NA	NA	NA	−.300	NA	NA	NA	NA	.585
Affective	NA	NA	NA	NA	NA	NA	NA	NA	NA	NA	NA	NA	NA	NA	NA	NA	NA	NA	.415	NA	NA	NA	NA	NA	NA	NA	NA	NA	NA	NA	NA	NA	NA	NA	NA	.172
Apathy	NA	NA	NA	NA	NA	NA	NA	NA	NA	NA	NA	NA	NA	NA	NA	NA	NA	NA	NA	NA	NA	NA	NA	NA	**.** ***507***	NA	NA	NA	NA	NA	NA	NA	NA	NA	NA	.257

Note: (1) The beta coefficients were noted. (2) The *p* value of all noted beta coefficients were less than 0.05, while the bold-faced italic values were less than 0.01. (3) Abbreviation: *NA* not available. *CASI* Cognitive Abilities Screening Instrument, *CDR* Clinical Dementia Rating, *HIS* Hachinski Ischemic Scale. *GCC* genu of the corpus callosum, *BCC* body of the corpus callosum, *SCC* splenium of the corpus callosum, *RSLF* right superior longitudinal fasciculus, *LSLF* left superior longitudinal fasciculus, *Rfminor* right forceps minor, *Lfminor* left forceps minor, *Rfmajor* right forceps major, *Lfmajor* left forceps major, *Ratr* right anterior thalamic radiation, *Latr* left anterior thalamic radiation, *Runc* right uncinate fasciculus, *Lunc* left uncinate fasciculus, *RILF* right inferior longitudinal fasciculus, and *LILF* left inferior longitudinal fasciculus
