# Supplementary material for: Comparison of neuropsychiatric symptoms and diffusion tensor imaging correlates among patients with subcortical ischemic vascular disease and Alzheimer’s disease
Source: BMC Neurol. 2017 Jul 28;17:144. doi: 10.1186/s12883-017-0911-5 (PMC5534111; doi:10.1186/s12883-017-0911-5)
Supplement: Supplementary file 1 — Cognitive function in standard scores (z score) among patient with Alzheimer’s disease. (DOC 47 kb) [file 12883_2017_911_MOESM1_ESM.doc]

**Additional file 1. Cognitive function in standard scores (z score) among patient with Alzheimer’s disease**

|  | All patients (*n* = 32) | CDR = 0.5  (*n* = 21) | CDR = 1 ~ 2  (*n* =11) |
| --- | --- | --- | --- |
| Cognitive Abilities Screening Instrument | -1.76 | -1.43 | -2.40 |
| Attention | -0.70 | -0.70 | -0.69 |
| Mental manipulation | -0.51 | -0.57 | -0.39 |
| Orientation | -2.29 | -1.33 | -4.12 |
| Short-term memory | -2.23 | -1.99 | -2.68 |
| Long-term memory | -0.19 | 0.15 | -0.82 |
| Language | -0.06 | -0.03 | -0.11 |
| Visual construction | -0.65 | -0.74 | -0.50 |
| Category fluency | -0.85 | -0.61 | -1.30 |
| Abstraction and judgment | -0.75 | -0.61 | -1.03 |
