# Supplementary material for: Comparison of neuropsychiatric symptoms and diffusion tensor imaging correlates among patients with subcortical ischemic vascular disease and Alzheimer’s disease
Source: BMC Neurol. 2017 Jul 28;17:144. doi: 10.1186/s12883-017-0911-5 (PMC5534111; doi:10.1186/s12883-017-0911-5)
Supplement: Supplementary file 2 — Intra-class correlation coefficients of measurement of diffusion tensor imaging parameters (0 ≤ fractional anisotropy ≤1; mean diffusivity: in units of m2 s−1 × 10^−9^). (DOC 45 kb) [file 12883_2017_911_MOESM2_ESM.doc]

**Additional file 2.** **Intra-class correlation coefficients of measurement of diffusion tensor imaging parameters (0 ≤ fractional anisotropy ≤ 1; mean diffusivity: in units of m^2^s^−1^×10^−9^).**

|  | Between-subject  ơ^2^ | Within-subject  ơ^2^ | Intra-class correlation coefficients | *p* | | 95 %  Confidence Interval |
| --- | --- | --- | --- | --- | --- | --- |
| **Fractional anisotropy** |  |  |  |  |  | |
| Genu | 2.024 | 0.105 | 0.95 | < 0.001 | (0.80, 0.98) | |
| Body | 2.046 | 0.105 | 0.95 | < 0.001 | (0.80, 0.98) | |
| Splenium | 3.225 | 0.105 | 0.97 | < 0.001 | (0.88, 0.99) | |
| **Mean diffusivity** |  |  |  |  |  | |
| Genu | 9.344 | 0.548 | 0.94 | < 0.001 | (0.02, 0.99) | |
| Body | 6.102 | 0.227 | 0.96 | < 0.001 | (0.03, 0.99) | |
| Splenium | 13.965 | 0.403 | 0.97 | < 0.001 | (0.03, 0.99) | |
